# Supplementary material for: Integration of Antifouling and Underwater Sound Absorption Properties into PDMS/MWCNT/SiO2 Coatings
Source: Biomimetics (Basel). 2022 Dec 18;7(4):248. doi: 10.3390/biomimetics7040248 (PMC9775102; doi:10.3390/biomimetics7040248)
Supplement: Supplementary file 1 [file biomimetics-07-00248-s001.zip › biomimetics-2084717-supplementary.pdf]

Integration of antifouling and underwater sound absorption properties into PDMS/MWCNT/SiO2 nanocomposites

Pan Cao[1]*, Huming Wang[1], Mingyi Zhu[1], Yifeng Fu[2]*, Chengqing Yuan[3,4]*

[1]College of Mechanical Engineering, Yangzhou University, Yangzhou 225127, China

[2]School of Automobile and Traffic Engineering, Jiangsu University, Zhenjiang 212013, China

[3]National Engineering Research Center for Water Transportation Safety, Reliability Engineering Institute, Wuhan University of Technology, Wuhan 430063, China

[4]School of Transportation and Logistics Engineering, Wuhan University of Technology, Wuhan 430063, China

Corresponding Authors: Pan Cao, E-mail: caopan@yzu.edu.cn

Yifeng Fu, E-mail: fu_yifeng@163.com; Chengqing Yuan, Email: ycq@whut.edu.cn

Supplementary information

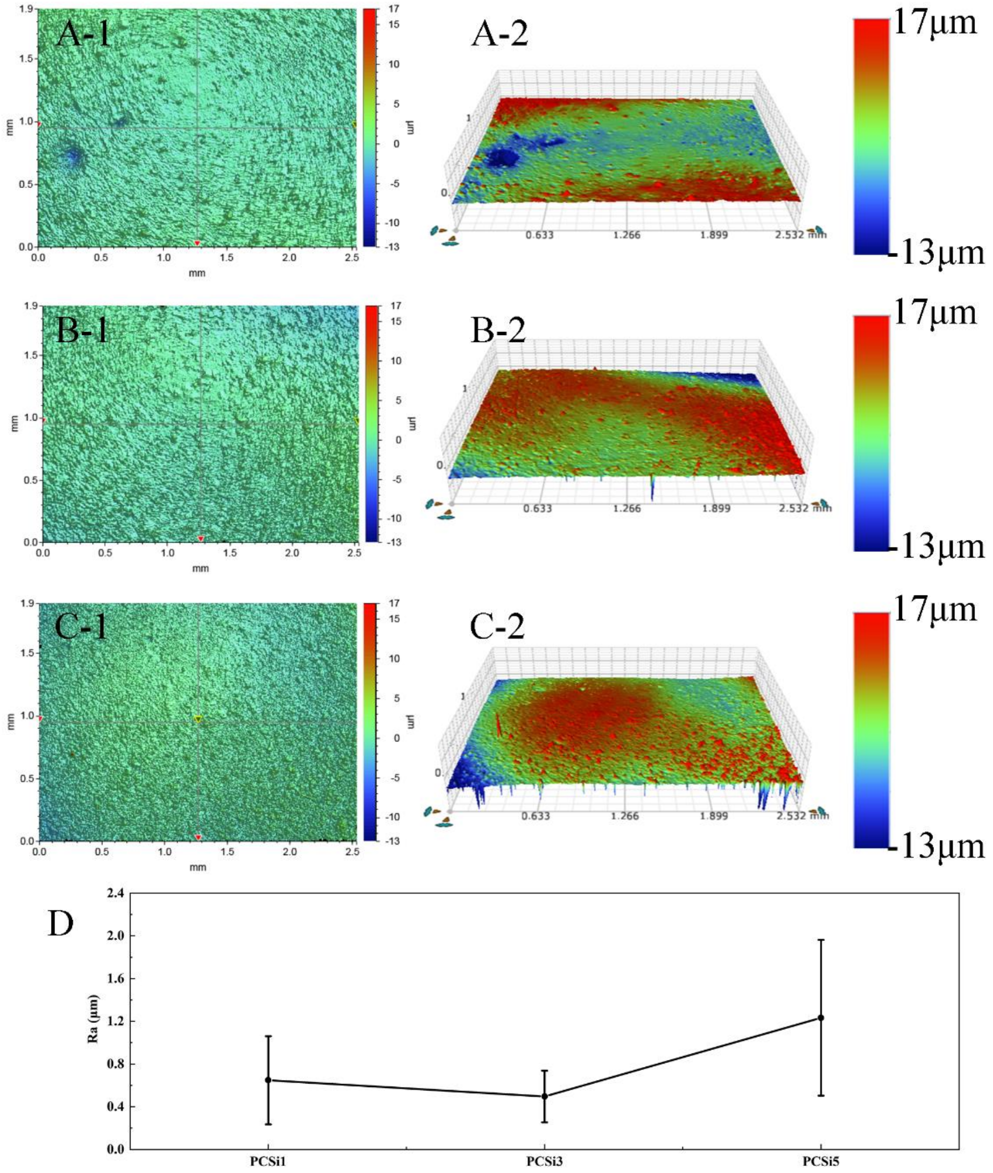

Figure S1. A-C: Surface profile of PCSi1, PCSi3, PCSi5. D: Surface roughness Ra.

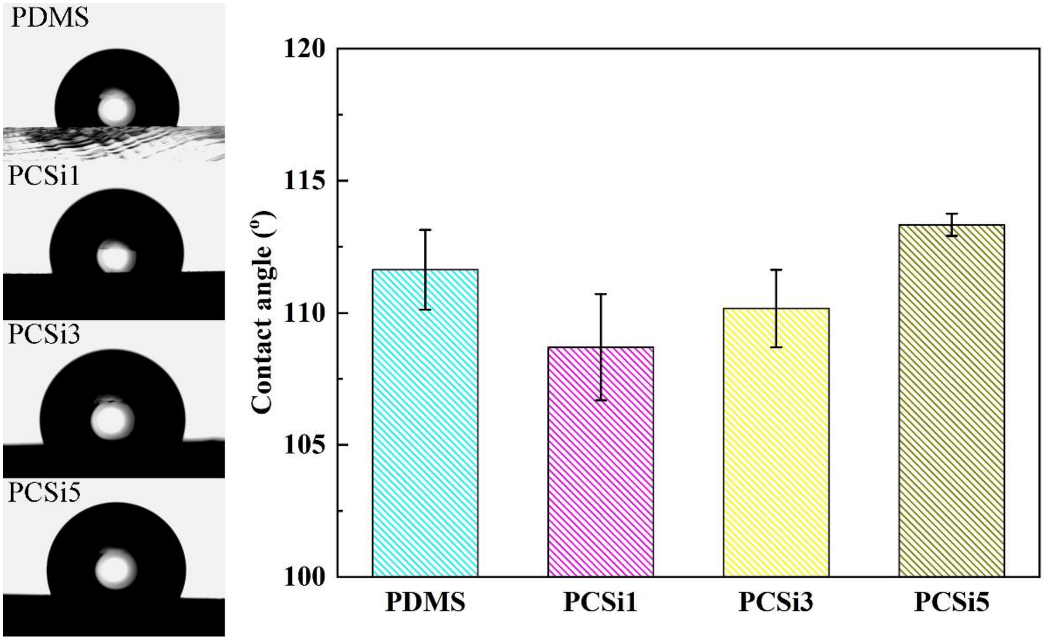

Figure S2. Contact angles of surface of different samples.

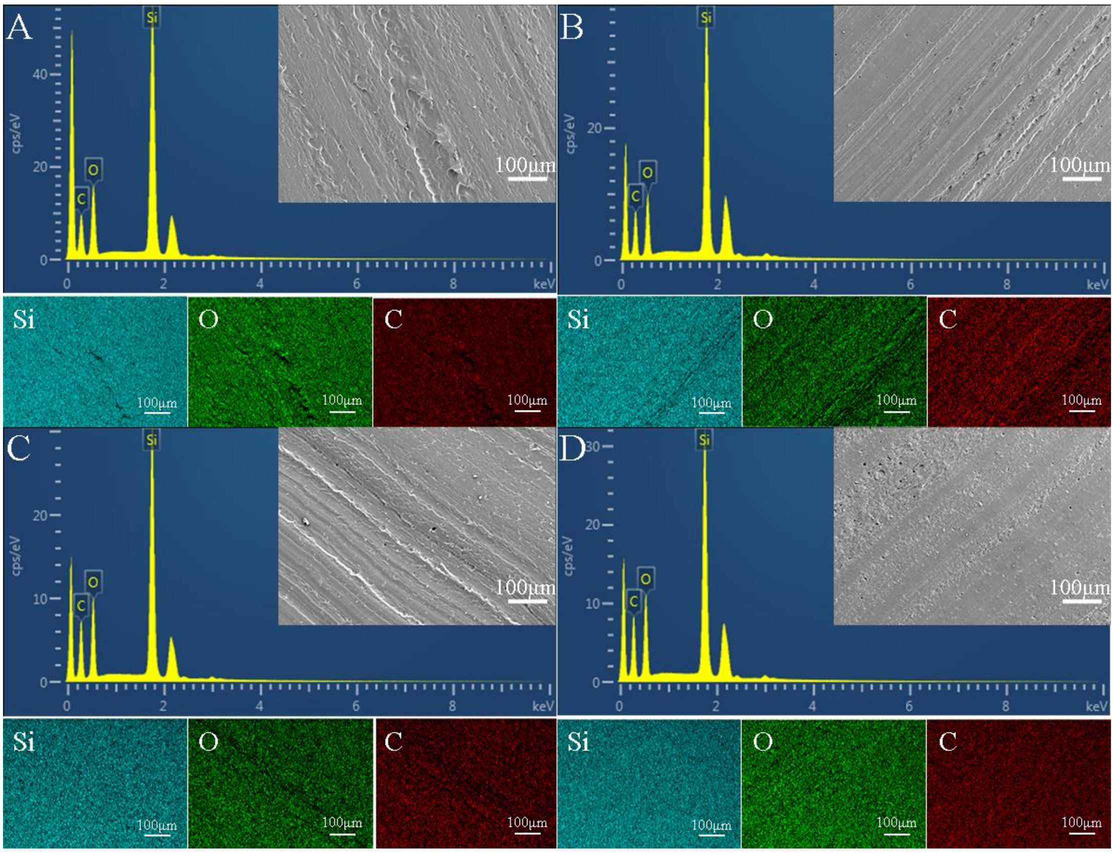

Figure S3. EDS analysis of PDMS (A), PCSi1 (B), PCSi3 (C), PCSi5 (D).

Table S1. The contents of different elements collected by EDS

Samples	C (wt%)	O (wt%)	Si (wt%)
**PDMS**	34.36	18.14	47.50
**PCSi1**	36.22	21.57	42.21
**PCSi3**	36.55	22.36	41.09
**PCSi5**	33.04	22.06	44.90
